# Editorial: Cryopreservation of mammalian gametes and embryos: implications of oxidative and nitrosative stress and potential role of antioxidants

**DOI:** 10.3389/fvets.2023.1174756

**Published:** 2023-04-12

**Authors:** Jones Ofosu, Yunhai Zhang, Ying Liu, Xiuzhu Sun, Guobo Quan, Manuel Alvarez Rodriguez, Guangbin Zhou

**Affiliations:** ^1^Key Laboratory of Livestock and Poultry Multi-omics, Ministry of Agriculture and Rural Affairs, College of Animal Science and Technology (Institute of Animal Genetics and Breeding), Sichuan Agricultural University, Chengdu, China; ^2^Anhui Province Key Laboratory of Local Livestock and Poultry, Genetical Resource Conservation and Breeding, College of Animal Science and Technology, Anhui Agricultural University, Hefei, China; ^3^Department of Animal, Dairy and Veterinary Sciences, Utah State University, Logan, UT, United States; ^4^College of Animal Science and Technology, Northwest A&F University, Yangling, China; ^5^Yunnan Animal Science and Veterinary Institute, Kunming, China; ^6^Department of Animal Reproduction, Instituto Nacional de Investigación y Tecnología Agraria y Alimentaria (INIA-CSIC), Madrid, Spain

**Keywords:** cryopreservation, reactive oxygen species, antioxidants, sperm, oocyte, embryo

## The effect of cryopreservation on gametes

Despite the advances in cryopreservation procedures, the developmental rate of frozen-thawed oocytes is still lower compared to their fresh counterparts. Mechanical damage by crystal ice formation, the toxicity of cryoprotective agents, osmotic pressure induced by low temperatures, and oxidative stress as a result of intracellular reactive species are the major cause of the reduced developmental capacity of the cryopreserved sperm and oocyte (1–4). Excessive generation of reactive oxygen species (ROS) and reactive nitrogen species (RNS) underline most cryoinjuries which ultimately retard basal functions and survival of gametes both *in vitro* and *in vivo* (3, 5). Key among the damages from stresses of cryopreservation on gametes is damage to the mitochondria and the endoplasmic reticulum (ER) which further leads to damage to the lipids, proteins, spindle, and DNA (2, 4).

## The main sources of ROS during cryopreservation

Cellular ROS during cryopreservation is genereated from two main sources, endogenous or exogenous, and is present as either the superoxide radical (${\text{O}}_{2}^{•-}$), hydrogen peroxide (H_2_O_2_), or hydroxyl radical (•OH). ${\text{O}}_{2}^{•-}$ is generally unreactive and has a short half-life (5). Gametes can remove it through the endogenous antioxidant system (3). Antioxidants such as superoxide dismutase (SOD) oxidize the ${\text{O}}_{2}^{•-}$ to H_2_O_2_ which binds to enzymes such as catalase and glutathione to produce water (H_2_O) and molecular oxygen (O_2_) through the Fenton reaction (4). However, a very reactive •OH is produced during this reaction that readily reacts with surrounding biomolecules and breaks them down eventually resulting in cellular damage (4, 5).

## Endogenous ROS during cryopreservation

Exogenous ROS is mainly generated from the environment and the reagents used in the experimental procedures. The main source of endogenous ROS is from the mitochondria but the ER and some oxidases can also generate ROS (5). Mitochondrial ROS is normally generated through the activities of the complexes I, III, and IV of the electron transport chain (ETC) which reduces O_2_ to H_2_O. ROS is however generated from about 1% of the O_2_ which is reduced to ${\text{O}}_{2}^{•-}$ due to electron leakage from the ETC which then rapidly generates H_2_O_2_ from –OH through the Fenton reaction and thus becomes the main site of intracellular ROS generation which is then released through the mitochondrial permeability transition pore (mPTP) into intracellular spaces where they can cause further ER stress (5). This is a result of Ca^2+^ overload which causes a continual opening of the mPTP leading to an increase in mitochondrial membrane potential (ΔΨ) and ATP dissipation. ROS from the ER is predominantly due to the release of Ca^2+^ into the cytoplasm through IP3R, particularly through the nicotinamide adenine dinucleotide phosphate (NADPH) oxidase 4 (NOX4) by Calcium/calmodulin-dependent protein kinase II (CAMKII) (4, 5). Some of the Ca^2+^ are absorbed by voltage-dependent anion channel (VDAC), an anion channel protein in the mitochondria leading to further generation of mitochondrial ROS through nitric oxide synthase (NOS) (5). Enzymatic activities of oxidases such as NADPH oxidase (NOX) and NOS in the cytoplasm and plasma membrane also generate ROS (6). This produces a self-sustaining endogenous ROS generation mechanism that decreases ATP levels and ΔΨ and causes oxidative damage to cellular lipids, proteins, and DNA in the sperm, oocyte, and embryo in porcine, mouse, and human oocytes (6–8). ROS from ER stress is produced by several enzymes such as protein disulfide isomerase (PDI), ER oxidoreductin-1 (ERO-1), and NOX4. PDI and ERO-1 transfer electrons to O_2_ and generate H_2_O_2_ through the flavin adenine dinucleotide (FAD)-dependent reaction. Alternatively, Nox4 uses NADH or NADPH as an electron donor to produce ${\text{O}}_{2}^{•-}$.

## Exogenous ROS during cryopreservation

Exogenous ROS is mainly generated from the environment and the reagents used in the experimental procedures. The sources include cryoprotective agents such as dimethyl sulfoxide (DMSO), extreme cold, light, pH of culture media, and metal ions (4). DMSO causes the release of excessive Ca^2+^ from the ER into the cytoplasm which is then uptaken by the mitochondrion contributing to mitochondrial ROS generation (9). Low temperature during cryopreservation induces ROS production in the presence of DMSO (9). Lights such as the blue light (400–500 nm) in the *in vitro* culture environment constitute radiation that generates H_2_O_2_ which alters enzymes in the respiratory chain (10). Ambient light such as ultraviolet B radiation (UVB) (290–320 nm) induces DNA base oxidation and strand breaking (10). Evidence suggests 5 min of exposure of mouse oocytes to visible light increases ROS levels (9). Higher pH values and O_2_ concentrations lead to higher oxidase activity and lead to elevated ${\text{O}}_{2}^{•-}$ levels (4). Fe^2+^ and Cu^2+^ metal ions in the *in vitro* media induce ROS production through the Fenton reaction and the Haber–Weiss reaction (11). In addition, Fe can also act directly on lipids and amplify peroxidative damage (4). Cardiolipin in the inner mitochondrial membrane releases cytochrome C which binds to apoptotic protein activator 1 (Apaf-1) and caspase-9 to activate the apoptotic pathway (11, 12). Under oxidative stress cis-aconitate which is an intermediate molecule of the mitochondrial tricarboxylic acid cycle (TCA) is inactivated, leading to Fe^2+^ and H_2_O_2_ formation, and causing oxidative metabolic dysfunction and decreased ATP production (12).

Laboratory practices can affect the concentraton of ROS that is generated. Gamete handling and cryopreservation protocols are crucial to post-thawed quality as they could influence the excessive production of ROS. Transport time and temperature are very crucial in reducing ROS build-up and oxidative stress (4). It has been demonstrated that ${\text{O}}_{2}^{•-}$ generated from ROS reaches peak accumulation at 3 h post-collection (13). This is attributed to xanthine oxidase and hypoxanthine accumulation during ischemia in sheep oocytes (14). Therefore, reducing transport time can greatly reduce ROS build-up. Furthermore, in bovine oocytes, high (above 40°C) and low (below 20°C) temperatures induce heat stress or cold shock which leads to intracellular ROS build-up and subsequently affects the quality of the oocytes and the development of the embryo (13, 14). The drastic changes in temperature during the cryopreservation and thawing process lead to Ca^2+^ influx and ROS accumulation in vitrified goat oocytes which reduces *in vitro* maturation (15). The selection of proper permeable and non-permeable cryoprotectants, vitrification with high cooling and warming rates, and addition of cytoskeleton relaxants or ice blockers into culture media can minimize oxidative stress to some extent (16).

## The nitrosative stress in gametes and embryos caused by cryopreservation

Nitrosative stress is a subset of oxidative stress that arises from an overproduction of RNS from the interaction of nitric oxide (NO) with oxygen-derived oxidants and includes nitrogen dioxide (NO_2_), peroxynitrite (ONOO–), and NO (3). RNS elicits modifications in several biomolecules through oxidation, nitrosylation, and nitration (17). Like ROS, mitochondria are the primary sites of RNS generation, particularly NO and ONOO– (3). NO forms ONOO– which further reacts with other molecules and forms NO_2_ and dinitrogen trioxide that causes mitochondrial dysfunction, inhibition of ATP production, DNA strand breaking, and triggers apoptosis through nitration and oxidation (18).

## Antioxidants as remedy to oxidative and nitrosative stress during cryopreservation

Gametes possess enzymatic antioxidants that maintain cellular homeostasis by preventing excessive ROS build-up, and oxidative and nitrosative stress. Notable among these antioxidants are superoxide dismutase (SOD), catalase (CAT), glutathione peroxidase (GPX), glutathione (GSH), cysteine (CYS), and cysteamine (CSH) (3, 19). These antioxidants prevent oxidative stress by either directly scavenging free radicals such as –OH and H_2_O_2_ or indirectly through secondary molecules. SOD catalyzes the initial detoxification of ${\text{O}}_{2}^{•-}$ followed by the conversion of the product into H_2_O by CAT or GPX (20). Indirectly, antioxidants act through antioxidant signaling pathways mostly through receptors (such as melatonin receptors, MTRs) to enhance GPX activity (21), or promote the expression of related antioxidant proteins such as MAD2, NRF1, NRF2, SIRT1, and Drp1 to reduce oxidative and nitrosative stress and improve post-thawed gamete quality [(8, 21–23); Qin et al.]. Antioxidant activities of these molecules are reported in human, bovine, ovine, porcine, and mouse gametes [(4, 24, 25); Qin et al.]. The endogenous antioxidant system is however not enough to offset the oxidative imbalance during cryopreservation (6). Several exogenous antioxidants have therefore found important usage in cryopreservation and are used at various stages including vitrification, warming, and/or culture media to mitigate oxidative and nitrosative stress, and maintain gamete quality and embryo development (6). These antioxidants include melatonin (24), quercetin (26), vitamin E (27), resveratrol (28, 29), L-carnitine (30), proline (31), coenzyme Q10 (32), and astaxanthin (25). Exogenous antioxidants however should be used with caution as studies show that beneficial effects are concentration-dependent but too high concentrations have a damaging effect on the gametes and embryos (4). In addition, a combination of antioxidants can be effective in maintaining post-thawed quality (33). For instance, the melatonin and resveratrol combination was more effective in reducing ROS levels in vitrified-warmed mouse GV-stage oocytes (33).

Gametes like many other cells form stress granules (SGs) when exposed to vitrification and warming which inhibit excessive ROS formation (34). SGs form part of the endogenous antioxidant system and are resistant to oxidative damage. The antioxidant activity is regulated by two core components, GTPase-activating protein SH3 domain-binding protein 1 (G3BP1) and ubiquitin-specific protease 10 (USP10) which act antagonistically (4, 34). Under physiological conditions, excessive G3BP1 inhibits the antioxidant activity of USP10 and elevates ROS steady-state. However, oxidative and nitrosative stress induce SGs formation which cause USP10 to induce ataxia-telangiectasia-mutated protein (ATM) phosphorylation which suppresses the inhibition of USP10 by G3BP1, thereby reducing ROS production and apoptosis (4, 34). Gametes have reduced ability to remove oxidized products than somatic cells probably due to the presence of high levels of unsaturated fatty acids making them prone to lipid peroxidation which is indicated by low GPX activity in mice sperm and testes when compared with other organs the lungs and blood (35).

## Conclusion

Extreme ROS concentrations generated from endogenous and exogenous sources affect the survivability of gametes and embryos via intrinsic and extrinsic apoptotic pathways characterized by mitochondrial ROS generation, lipid peroxidation of polyunsaturated fatty acids-rich sperm membrane, DNA damage, and loss of ATP. Endogenous and exogenous antioxidants can offset this ROS imbalance and improve gametes and embryo quality and development in a concentration-dependent manner. Improved protocols, limiting handling time and exposure to bright light could reduce excessive reactive species build-up and improve gamete and embryo quality. Understanding of ROS and RNS and their impact on cryopreserved gametes continue to be broadened but many more studies are needed to still improve cryopreservation procedures because the quality of post-thawed gametes is still low when compared with fresh ones.

## Author contributions

JO: literature collection and review and writing—original draft. GZ: discussion and proofreading, supervision, and project administration. All authors approved the final version of this editorial.
